# Chronic Morel‐Lavallée Lesion Treated With Percutaneous Ultrasonic Debridement

**DOI:** 10.1002/jcu.70082

**Published:** 2025-09-16

**Authors:** John D. Karp, Samuel O. Oduwole, Levon N. Nazarian

**Affiliations:** ^1^ Department of Radiology Hospital of the University of Pennsylvania Philadelphia Pennsylvania USA; ^2^ Department of Physical Medicine and Rehabilitation Hospital of the University of Pennsylvania Philadelphia Pennsylvania USA

**Keywords:** Morel‐Lavallée, Tenex, tenotomy, ultrasound

## Abstract

Morel‐Lavallée lesion is defined as an internal degloving injury. A trauma involving shearing forces causes separation of the superficial fascia from the overlying subcutaneous tissue and skin, creating a potential space for hemolymphatic fluid to accumulate. This leads to the development of a self‐resolving or potentially chronic collection. Classically, lesions arise in the lateral thigh. Imaging features depend on the duration of the lesion. Acutely, ultrasound and MR imaging will demonstrate a hematoma‐like mass that is parallel to the underlying fascia. As the lesion evolves over time, calcifications with a dense capsule can form. Patients typically present with a persistent, localized swelling and pain that may radiate throughout the thigh. Initially, conservative therapy can be efficient; however, once a capsule forms, surgical management is the current gold standard therapy. Tenex (Tenex Health, Lake Forest, CA) is a device that uses high‐frequency ultrasonic waves to perform percutaneous debridement to disrupt and remove calcifications in the setting of various chronic musculoskeletal conditions. This case describes the successful use of this technology in a 68‐year‐old female with a 10‐year history of a Morel‐Lavallée lesion. Treatment of Morel‐Lavallée lesions using the Tenex device is a feasible, minimally invasive alternative to conventional surgery.

## Graphical abstract

This case describes the use of Tenex in a 68‐year‐old female with a 10‐year history of a Morel‐Lavallée lesion. The device uses high‐frequency ultrasonic waves that percutaneously debride dystrophic musculoskeletal calcifications. Percutaneous ultrasonic debridement is a minimally invasive intervention to be considered for managing symptoms associated with chronic Morel‐Lavallée lesions.

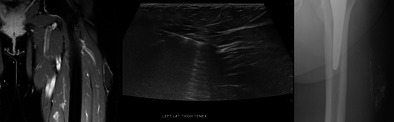

## 1 Introduction

Morel‐Lavallée lesion, first described by Dr. Victor‐Auguste‐François Morel‐Lavallée in 1863, is a degloving injury arising from traumatic shearing forces (Morel‐Lavallée 1863). Acutely, a fluid‐filled cavity is formed in the prefascial plane after separation of the subcutaneous tissues from underlying fascial layers. Lesions may resolve spontaneously; however, inflammation may lead to a fibrous capsule over time, becoming chronic. The fibrous capsule may calcify (Singh et al. 2018; McKenzie et al. 2016). Morel‐Lavallée lesions can be secondary to various traumatic mechanisms such as blunt, crush, or high‐energy forces. Nearly 25% of patients with these lesions have been involved in a traffic accident (Shen et al. 2013). Males are twice as likely to be afflicted by these lesions than females (Lecky et al. 2017; Bonilla‐Yoon et al. 2014). Lesions usually present clinically as a tender unilateral enlarging mass over the greater trochanter after injury, but other common areas include the knee, abdomen, and buttock. Although MRI is the imaging modality of choice for diagnosing these lesions, ultrasound has a central role in image‐guided intervention. Acute Morel‐Lavallée lesions may be managed conservatively with compression bandaging. Needle aspiration and/or sclerosing agent infiltration can be offered for further therapeutic benefit. Surgical management is indicated for chronic or recurring lesions usually after the fibrous capsule has formed. Unfortunately, there is no current alternative for poor surgical candidates or patients unwilling to undergo surgery. We therefore present a case utilizing a tool designed to perform percutaneous ultrasonic debridement to treat a 68‐year‐old female with a 10‐year history of a Morel‐Lavallée lesion (Singh et al. 2018; Shen et al. 2013; Tenex Health 2020).

## 2 Case Report

**FIGURE 1:**
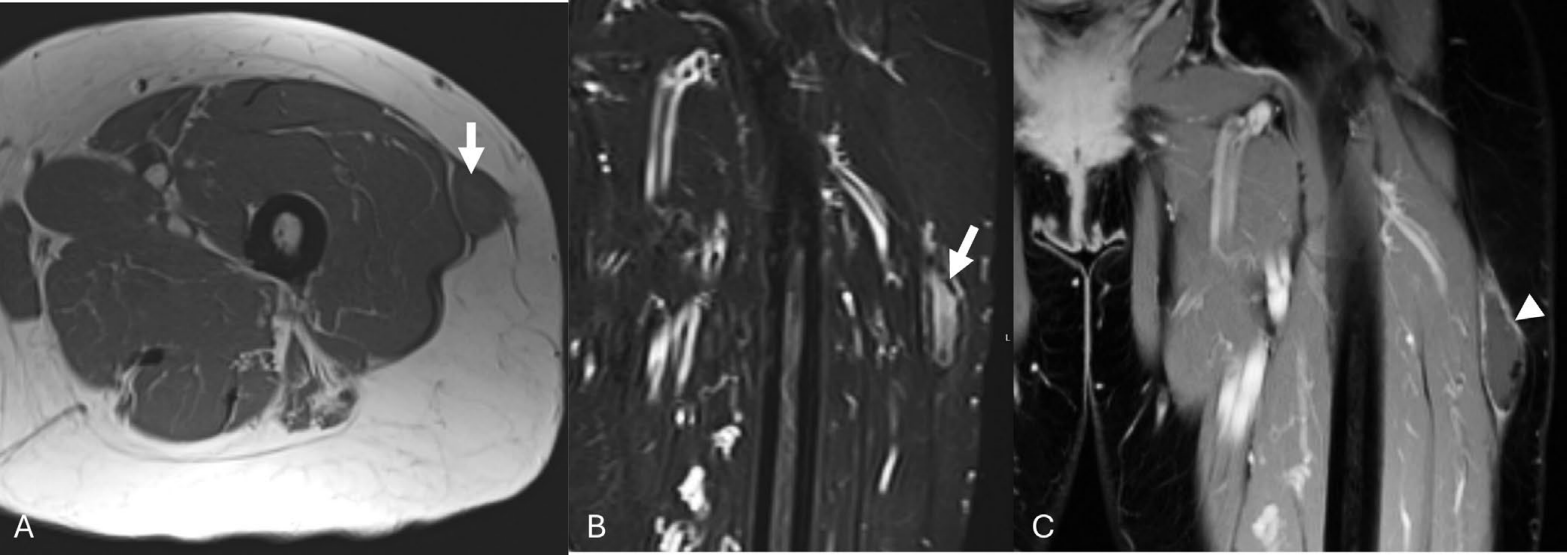
Initial MR axial T1 (A), coronal STIR (B), and post‐contrast coronal T1 (C) images demonstrating a T1 hypointense, T2 hyperintense lenticular‐shaped lesion measuring 5.7 × 1.5 × 3 cm (Tall × AP × Trv) with very hypointense peripheral components reflecting calcifications (arrow). T1 weighted post‐contrast imaging exhibits mild peripheral enhancement (arrowhead).

A 68‐year‐old female patient presented to her primary care physician 9 years ago with a left thigh mass noticed a year prior. Her exam was notable for a minimally tender, firm, fixed, and irregular lateral mass with no associated neurological deficits. MR imaging characterized a 5.7 × 1.5 × 3 cm (craniocaudal × anterior posterior × transverse) lesion with T1 isointensity and T2 hyperintensity to skeletal muscle (Figure 1). Peripheral low‐signal intensity on T1 and T2‐weighted imaging was consistent with hemosiderin or calcification, and post‐contrast imaging demonstrated mild peripheral enhancement. Deliberation of the case in the tumor board led to a consensus of Morel‐Lavallée as the likely diagnosis, so no tissue sampling was obtained.

**FIGURE 2:**
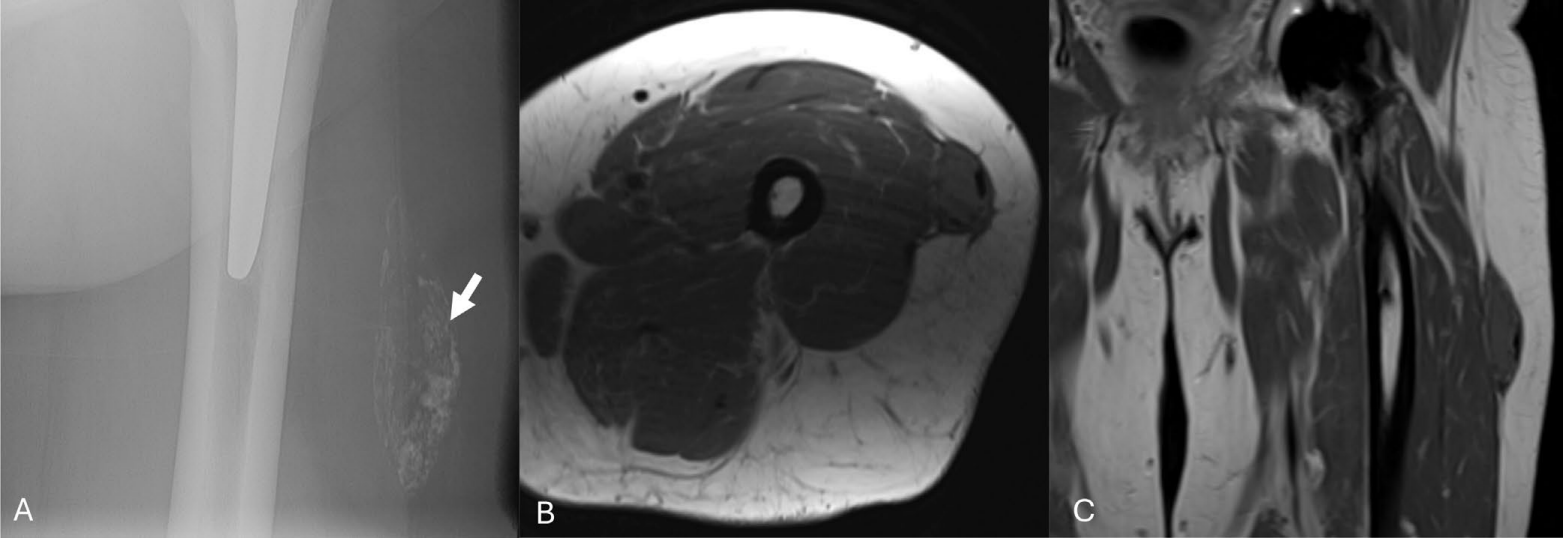
Preprocedural left thigh radiograph (A) and MR axial T1 (B), coronal T1 (C) images. The radiograph demonstrates a partially calcified ovoid mass within the left lateral thigh soft tissues (arrow). The subsequent MR images demonstrate a near identical lesion measuring 6.5 × 1.9 × 3.0 cm, an increase of 0.8 × 0.4 × 0 cm in 7 years.

**FIGURE 3:**
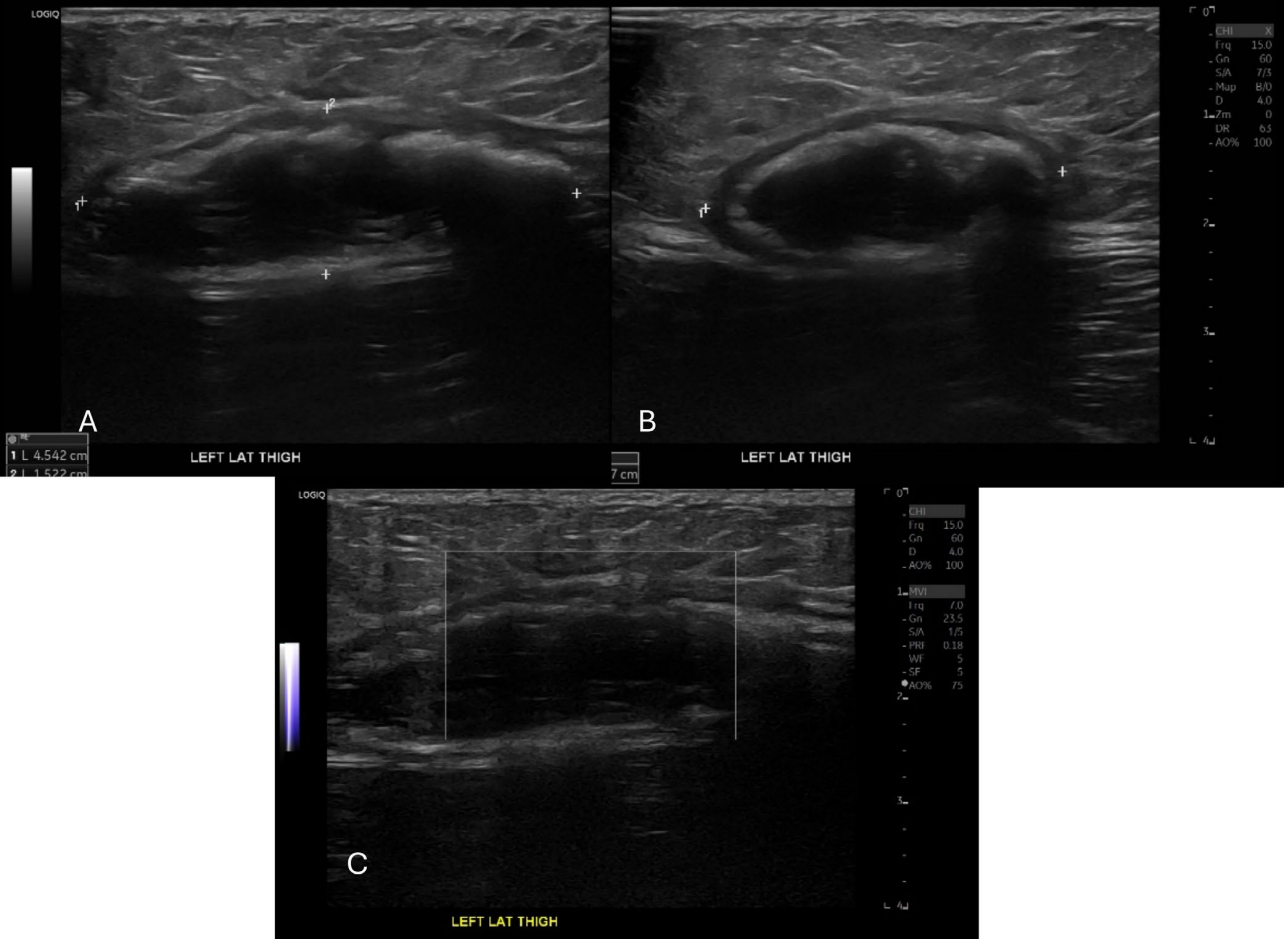
Pre‐procedural ultrasound images in long‐axis (A), short‐axis (B), and with power Doppler (C). The lesion had a coarse, partly calcified hyperechoic capsule, internal hypoechoic material with hyperechoic debris, and demonstrated no flow on Doppler imaging. The preprocedural measurement of the lesion was 5.9 × 1.5 × 3.3 cm.

Seven years later, the patient underwent bilateral total hip arthroplasty for severe osteoarthritis complicated by a motor vehicle accident with progressively worsening new left thigh pain at the site of the mass. Postoperative radiographs and MR imaging demonstrated a near identical 6.5 × 1.9 × 3.0 cm mass within the soft tissues (Figure 2). Medical management was unsuccessful, so the patient was referred for treatment with the Tenex device (Tenex Health, Lake Forest, CA) with the TX‐Bone attachment. Preprocedural ultrasound images demonstrated a 5.9 × 1.5 × 3.3 cm mass with a coarse calcified hyperechoic capsule, internal hypoechoic material with hyperechoic debris, and no flow on Doppler imaging (Figure 3).

**FIGURE 4:**
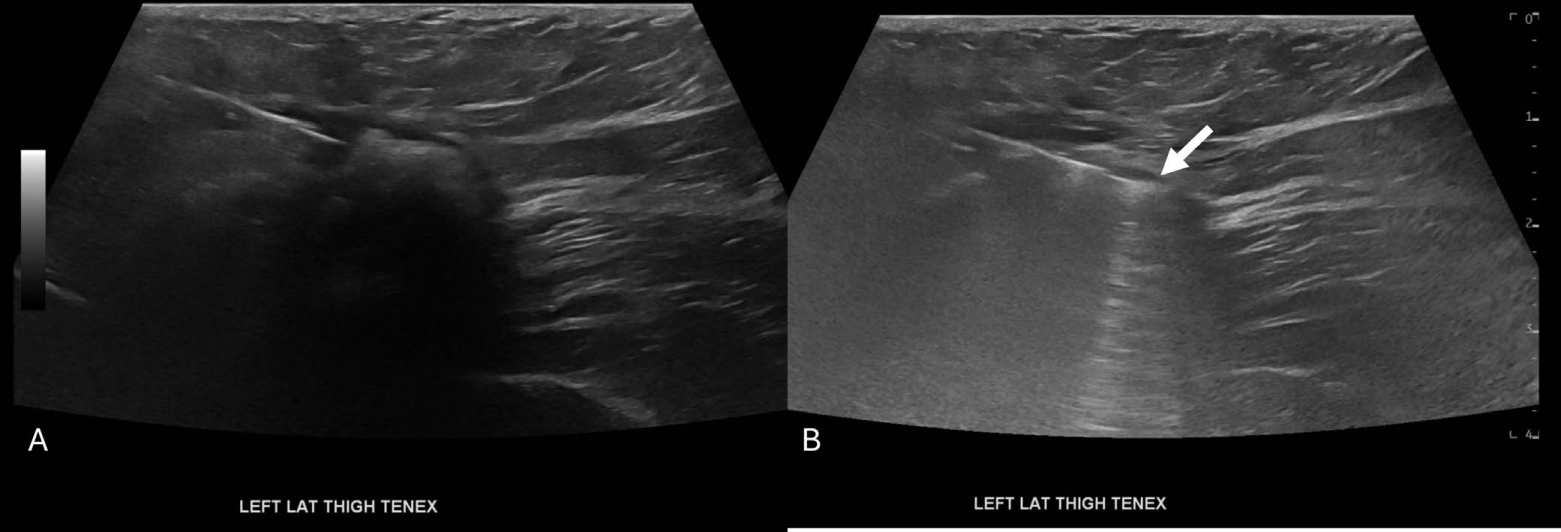
Intra‐procedural ultrasound image of the intralesional Tenex needle tip. The needle is seen within the lesion (A) before activating the ultrasonic tenotomy pulse waves demonstrating ring‐down artifact (B) (arrow) to disrupt the outer calcified capsule.

Patient was prepped and draped in the usual sterile fashion. A sterile sleeve was placed over the ultrasound transducer. After inserting the local anesthetic needle into the lesion, no fluid could be aspirated from within the lesion prior to the administration of 1% lidocaine. An #11 blade was used to incise the skin. Under continuous ultrasound visualization, the TX Bone handpiece was introduced through the incision. Once the tip of the instrument was confirmed within the calcified Morel‐Lavallée lesion, the foot pedal was depressed and the lesion was debrided percutaneously (Figure 4). The procedure was concluded at approximately 8 min when the peripheral calcified capsule had become considerably less dense sonographically and on palpation. The TX Bone handpiece was removed and hemostasis maintained. Following the procedure, a dressing was applied over the area. The patient reported 0/10 pain immediately afterward, a change from 9/10 prior.

**FIGURE 5:**
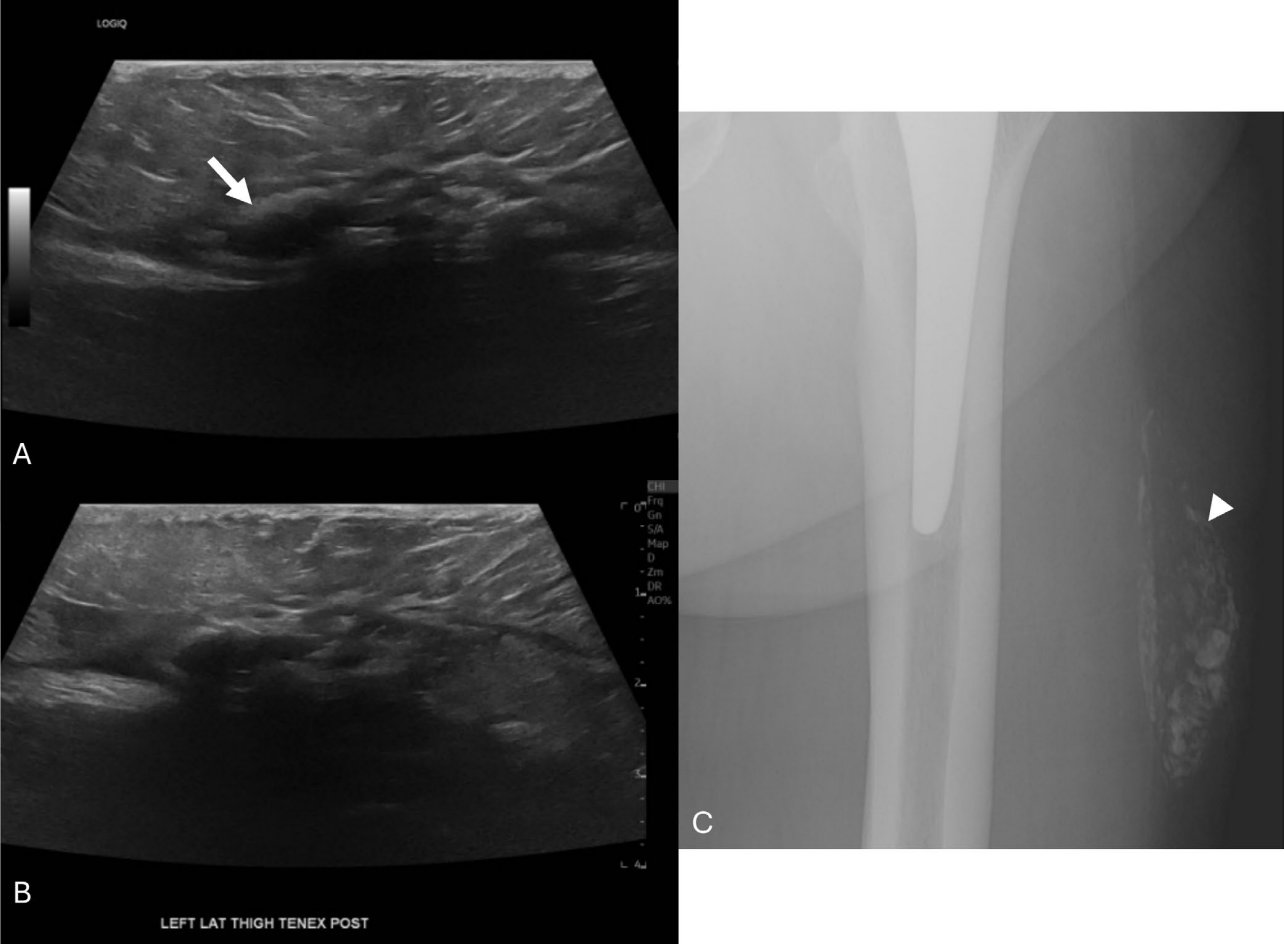
Postprocedural ultrasound images in long‐axis and short‐axis (A, B). Following the procedure, the lesion is flattened, the capsule is less echogenic, and the internal material has decreased in bulk. Postprocedural measurement was 4.9 × 1.0 × 2.9 cm, an overall decrease of 1.0 × 0.5 × 0.4 cm. The radiograph (C) shows a similar‐appearing calcified left lateral thigh mass that decreased in size by 0.9 × 0.4 cm (Tall × Trv).

Postprocedural ultrasound images showed flattening of the mass measuring 4.9 × 1.0 × 2.9 cm, an immediate decrease of 1.0 × 0.5 × 0.4 cm (Figure 5). The postprocedural radiograph grossly looked similar, with a decrease of 0.9 × 0.4 cm compared to the preprocedural radiograph.

The patient noted a marked improvement in pain and discomfort at her next orthopedic appointment a week later. At the 3‐month follow‐up, the patient reported being satisfied with the procedure and reported no pain or swelling at the site of the mass.

## 3 Discussion

Morel‐Lavallée lesions are uncommon injuries resulting from high‐energy shearing forces. The space created between the subcutaneous tissue and underlying fascia, commonly in the lateral thigh, can create localized, persistent swelling that results in difficult‐to‐treat pain. Once the lesion develops a capsule, treatment becomes limited to surgical intervention. Tenex is a novel device that has proven successful in minimally invasive debridement of tendinopathy (Barnes 2013). This device uses high‐frequency ultrasonic waves to simultaneously debride, emulsify, and aspirate the abnormal tissue. Tenex is emerging as a useful tool for percutaneous tenotomy, debridement, and ultrasonic fasciotomy in cases of common extensor tendinopathy, rotator cuff tendinopathy, plantar fasciopathy, and patellar tendinopathy, among others (Barnes 2013; McMullen and Liem 2018). In addition, Tenex has been described as safe, with reports of complications as being mild and rare (Fick et al. 2021).

This is the first reported use of this device in treating a Morel‐Lavallée lesion. This lesion became symptomatic 7 years after its initial discovery. In addition, there was a lack of significant change in the MRI appearance over this time. The rationale for delayed onset tenderness is unknown and likely multifactorial. There was a marked immediate decrease in pain and lesion size on ultrasound and radiography after intervention by Tenex. The patient reported sustained pain relief at the 3‐month follow‐up. Current literature suggests that Tenex patient reported pain relief is seen as early as 1 month and is sustained for at least 12 months (Parry et al. 2024; Erickson and Jagim 2020; Maag et al. 2024), even in patients where improvements in imaging appearance were modest (Trivedi et al. 2024). The Tenex treatment time of 8 min in our patient compares favorably with the mean times of 5 ± 1.7 min in a study reporting favorable treatment of calcific tendinopathy in the shoulder (Erickson and Jagim 2020).

Treatment of Morel‐Lavallée lesions can be broken down broadly into surgical and non‐surgical management. The non‐surgical options include compression bandaging, percutaneous aspiration, or sclerodesis and are typically utilized in the acute phase (Shen et al. 2013). Conservative management and percutaneous aspiration have a reported recurrence rate of 56% (Vess et al. 2023). Sclerodesis involves draining and infiltrating the lesion with doxycycline, erythromycin, or bleomycin to induce cell destruction and fibrosis of the surrounding tissues. The reported success rate of sclerotherapy for chronic Morel‐Lavallée lesions is up to 95.7% (Yang and Tang 2023). Current mainstay management for chronic lesions is surgery. Surgical management involves open drainage and mass resection with primary or secondary closure, with or without drain placement. (Shen et al. 2013; Lecky et al. 2017) The more aggressive treatment modalities offer the highest efficacy, especially in recurrent cases with multiple treatment failures.

## 4 Conclusion

Based on the favorable outcome of this case, we believe that percutaneous ultrasonic debridement should be considered for patients with chronic lesions who have failed conservative management and/or who are not surgical candidates. While surgery is an effective approach for chronic Morel‐Lavallée lesions, the risks may outweigh the benefits, especially when an alternative such as debridement with the Tenex device is available.

## Author Contributions

**John D. Karp:** writing – original draft, writing review, and manuscript editing. **Samuel O. Oduwole:** writing the original draft, writing the review, and editing the manuscript. **Levon N. Nazarian:** conceptualization, manuscript oversight, and manuscript editing.

## Ethics Statement

The use of the individual's depersonalized medical information was approved and informed consent was obtained from the patient.

## Conflicts of Interest

Levon N. Nazarian is on the Medical Advisory Board for Tenex (Unpaid). The other authors declare no conflicts of interest.

## Data Availability

The data that support the findings of this study are available from the corresponding author upon reasonable request.
